# Some Challenges for the Human Brain in Communication With the Digital Society

**DOI:** 10.3389/fpsyg.2020.594941

**Published:** 2020-10-27

**Authors:** Ana María Ruiz-Ruano García, Jorge L. Puga

**Affiliations:** ^1^Departamento de Psicología Evolutiva y de la Educación, Facultad de Ciencias de la Educación, Universidad de Granada, Granada, Spain; ^2^Departamento de Personalidad, Evaluación y Tratamiento Psicológico, Facultad de Psicología, Universidad de Granada, Granada, Spain

**Keywords:** social networks, mental health, digital society, education, communication

We cannot live or even exist without communication. Since we are social beings, we are unquestionably affected and modulated by communicative processes. Although we are shaped by communicative influences, we are also able to affect our environment just by communicating. In a sense, we could say we are human beings because we communicate our feelings, emotions, thoughts, or reasons. At the same time, we are currently experiencing drastic changes in communication methods. It seems our ways to communicate are changing from a face to face basis to a digital one. We are living in a globally and digitally interconnected world. Take Facebook or WhatsApp as examples. It was estimated that, in the first half of 2020, Facebook and WhatsApp had 2.5 and 2.0 million users, respectively (www.statista.com). These figures are even higher if we consider that COVID-19 pandemic has forced the world in quarantine which has contributed to an increased usage of communication digital tools. For example, the elderly living in retirement homes have adapted to use digital devices for communicating with their relatives, something unthinkable for some of them a few months ago.

If estimations are correct, the number of digital bytes will soon surpass the number of stars in the known universe (Butler, 2016). Roughly speaking, the amount of digital information grows exponentially and the capability of computers doubles every year. Although our brain can easily adapt to this massive amount of electronic data considering its phylogenetic evolution (Dehay and Kennedy, 2020), we think this situation raises some challenges when conceptualizing the brain embedded in a digital society. In our view, the potentially unreliable and massive amount of available digital information is a threat to the human brain. Firstly, this overload of information challenges our brains because our cognitive system is limited at some levels of information processing (Sweller, 2020). On the other hand, potentially unreliable, confusing, or even contradictory information is able to destabilize the human brain. We are not complaining about technological evolution nor to the production of massive digital data, but we would like to note some issues deserving careful attention when making a transition to a healthier and safer digital generation. Although we agree that our current digital society has the potential to provide us with information to solve long lasting social problems (Ledford, 2020; Shah, 2020), we have also to be aware that the technology we use for that purposes might be biased and not shifting the power status-quo generating those problems (Courtland, 2018; Kalluri, 2020; Saltelli et al., 2020). As a result, we would like to highlight some topics we think deserve some consideration to help future generations not to struggle with the social impact of digital communication.

Consider, for example, the smartphone misuse. Although phone apps have been developed to positively treat mental health problems (Abbot, 2016; Anthes, 2016), some patterns of smartphone social interaction are also considered risky to mental health (Ruiz-Ruano et al., 2020). For example, messages and notifications in digital social networks are supposed to play a critical role in the development of psychological disorders (Veissière and Stendel, 2018). As a result, some guidelines are needed to recommend users how to healthily interact with their mobile phones and how to manage the overwhelming amount of information they are exposed to.

Digital social interaction is also a critical issue when considering information and news spread. We can now share pieces of information with our acquaintances in a matter of seconds. Our contacts in social networks can also share this information with their acquaintances and so on. From a network point of view, the spread of this kind of information is governed by a small-world rule which means that a single piece of information is able to reach the other side of the world in just a few jumps (Milgram, 1967, 1969). This possibility is nowadays considered to be amazing because it, let us say, democratizes information propagation. However, it also has some drawbacks. For example, it seems that false news propagates quicker than true ones. Vosoughi et al. (2018) observed that falsehoods are 70% more likely shared in social networks than true pieces of news. They also noted that false news is considered more novel by social network users and this novelty pushes this kind of news further. This preference for novel news, theories, and explanations is also pervasive in scientific grounds (Antonakis, 2017). As a result, the spread of false or potentially damaging news is extremely fast in small worlds like digital social networks. This propagation mechanisms reminds infectious diseases spread (Watts and Strogatz, 1998) and the term “infodemics” has been coined to refer to this potentially dangerous phenomenon (Ball and Maxmen, 2020).

Social digital networks evolve over time. This evolution has also been described in mathematical terms. One of the best-known basic mechanism of network evolution is that one described by the concept of preferential attachment and the statement “the rich get richer” (Barabási and Albert, 1999). When considering information propagation, this refers to what we call “super-spreaders” in the context of infectious diseases. This means that some nodes in social digital networks act as special hubs hyper-propagating falsehoods through the web. Imagine someone joining to a digital social network platform. This new node in the network will tend to link to previously existing nodes. On average, new digital social networks users will attach to nodes with more connections. For example, new users tend to attach to those known as “influencers” which might serve to spread information at a higher rate. In case of potentially false information, influencers become super-spreaders of potentially false news.

One of the risks we are facing when considering communication in digital social networks is what has been named as the hybrid warfare (Hoffman, 2009; Ducaru, 2016; Lanoszka, 2016). This kind of conflict is seen as a type of war in which classical and overly physical violence is replaced by an implicit attack to rightfully consolidated societies. A type of conflict in which the social organization is challenged by a mixture of fake propaganda aimed at destabilizing governments and stable democracies (Lafuente, 2015). Some authors suggest that this modus operandi is expected to be more frequent, sophisticated, and destructive in the future and our digital society is relatively vulnerable to those types of attacks (Taddeo and Floridi, 2018). From a technical point of view, what is threatened is not data integrity but information integrity. That is to say, the reliability and trustworthiness of information (Von Solms and van Niekerk, 2013). As we pointed out above, sharing, and labeling news in digital social networks might help to propagate false news generating social conflict and destabilizing peaceful, rightful, and legitimate societies.

Another critical point is education. In our opinion there are at least two focus of concern for a digital society at this level. The first concern is related with the availability of information and how the students access, manage, filter, and use information. We lecture at university and we are used to see plagiarized academic projects (Puga, 2014). When verbatim plagiarism is not present, the produced text in academic projects can be considered as a collage of rephrased text coming from the web. It seems that students are overwhelmed by the amount of available information on the web and they are unable to filter and manage the information they access properly. By the way, some recent research have noted potential problems for learning when reading from screens instead of physical pages (Mangen et al., 2019; Støle et al., 2020). Additionally, students seem to blindly trust on searching engines to find answers and solutions to their academic problems. We think this behavior might be problematic in case it is systematically repeated by students. In the long run, we are afraid of seeing our students asking a searching engine the question “who am I?” to write a piece of homework for the philosophy class. We are not criticizing destructively the potential of digital information for educational purposes. On the contrary, technology has provided us with lots of tools to teach-and-learn almost anything independently of the country we are living, our race, gender, social class, or income (Waldrop, 2013). Take for example the Massive Online Open Courses (MOOCs). Those courses are designed to provide digital opportunities for learning and teaching in a free-and-open fashion. However, we wonder whether digital social interaction inside those courses is as fruitful as the interaction expected and observed in in-person campuses. And, what is more, as suggested by Emanuel (2013), we also worry those courses are not reaching the part of the population in real need of it.

Social interaction in a digital society is not negative *per se*, but this form of communication challenges the world and individuals (brains) in several ways. The amount of available digital information appears to be critical for human beings in several ways. If our brain is considered a cognitive system with a limited capacity of processing, we should take actions to prevent the system to be overloaded to avoid failure. We have noted some critical points in which overload should be prevented: mental health, information sharing in social networks and education. We think these three dimensions are essential in our digital society and we should think carefully about it to advance toward a healthier and safer digital society. To prevent mental health problems related with digital social interaction we suggest carrying out more research to identify risk factors to develop mental disorders in connection with virtual communication. The “infodemic” threat should be addressed avoiding censorship practices and promoting high quality journalism searching for the truth. Maybe a fact checked-based naïve journalism can be promoted into individual users of social networks to break the chain of falsehood propagation. At educational level, we suggest providing students with competences to search, filter, and use high quality online documentation. Unless we help our students to assess critically the sources and content of the information they use, we will not be able to prevent them cheating from the web. Training on synthetic writing is also critical at this level.

Technological progress is probably a stepped function in history because technical advances shake humanity from time to time and generate new paradigms of living. Along history, wealth, and the important things to live have been understood differently. Today, having and constantly using a digital device per person (let's say for example a mobile phone, laptop, or tablet) to communicate seems to be the in-thing. Nevertheless, we forget that there are also important things we cannot do virtually, for example eating or caring for health. An old person in our close context once said that: “a computer or mobile phone cannot harvest potatoes,” referring to the limited usefulness of the information and communication technologies. The interaction with digital devices helps our minds to improve some cognitive skills. These devices help us to accomplish tasks we cannot do otherwise. We can keep in contact with relatives, friends, or workmates. We can also share ideas, emotions, and information all over the world in a matter of seconds. However, when abusing of this type of social interaction we are not practicing and missing other activities that people have done face-to-face along the history. Kissing, hugging, and smelling your mother, father, child, or partner is something we cannot do with a computer or, at least, it does not taste the same. The time information and communication technologies are with us is insignificant if we consider the humanity history. In that sense, we need to monitor and prevent the negative impact of digital information in our lives and environment. Specially because this impact is still unknown. That is the reason why we have provided some issues to reflect on. We hope it can be helpful for others to think and prevent the negative and unplanned impact of digitalization on humanity and the human brain.

## Author Contributions

All authors conceived, discussed, and wrote the paper. They contributed equally to the development of the manuscript.

## Conflict of Interest

The authors declare that the research was conducted in the absence of any commercial or financial relationships that could be construed as a potential conflict of interest.
